# Study on the Correlation between Continuity of Care and Quality of Life for Patients with Coronary Heart Disease

**DOI:** 10.3390/ijerph17239125

**Published:** 2020-12-07

**Authors:** Hsiang-Chu Pai, Yi-Fang Hu, Shu-Yuan Chao, Hsiao-Mei Chen

**Affiliations:** 1 Department of Nursing, Chung Shan Medical University, Taichung 40201, Taiwan; pai55215@csmu.edu.tw; 2 Department of Nursing, Kuang Tien General Hospital, Taichung 43303, Taiwan; cgh123654@yahoo.com.tw; 3 Department of Nursing, Hungkuang University, Taichung 43302, Taiwan; curie_chao@hotmail.com

**Keywords:** coronary heart disease, continuity of care, quality of life, instrumental activities of daily living

## Abstract

*Background*: As coronary heart disease (CHD) is a highly complex disease, complex continuity of care (CoC) service should be provided for the patients, and the quality of life (QoL) needs to be regarded as an important measuring indicator for the health-care outcome. *Purpose*: To understand the general situation of CHD QoL and important predictors. *Method:* A cross-sectional study design was adopted from August 2019 to July 2020 by structured questionnaires. A total of 163 patients were enrolled, and data were statistically analyzed using SPSS 25.0. *Result:* The average score of the QoL questionnaire is 56.56/80, and the CoC is 4.32. The overall regression model can explain 58.7% of the variance regarding QoL. Patients’ instrumental activities of daily living (IADLs) (26.1%), age (18.1%), living situation (7%), information transfer (4.8%), main source of income (1.8%), and risk of disability are significantly different from their overall QoL in depression (0.9%). Conclusions: In order to improve the QoL of patients, it is suggested that medical teams should assess the needs of patients immediately upon hospitalization, provide patients with individual CoC, encourage them to participate in community health promotion activities, and strengthen the function of IADL to improve the QoL of patients.

## 1. Introduction

Health-related quality of life issues for coronary heart disease (CHD) patients has always been a topic of concern and attention in clinical health care [1]. The most important risk factors for CHD include men, age (>45 years), post-menopausal women, obesity, smoking history, lack of physical activity, and related chronic diseases [2]. Disability, which covers injuries, activity restrictions, and participation restrictions, affects approximately 41% of CHD patients in the United States [3,4]. CHD patients will lose disability-adjusted life-years (DALYs) due to disability, resulting in an increase in the global burden of the disease (GBD) and affecting the quality of life (QoL) of patients [4]; thus, it is necessary to prevent the risk factors of CHD patients in clinical practice. Those with severe disease will have dysfunction in daily activities, including the activities of daily living (ADL) and instrumental activities of daily living (IADL) [5,6], and patients also often suffer from psychological distress [7], resulting in anxiety, depression, stress, or frustration [8].

Continuity of care (CoC) is a patient-centered health-care service that provides patients with correct and consistent medical information through cross-team cooperation to ensure that patients can receive seamless care, have confidence in the medical team, receive emotional support, and enjoy improved QoL [9]. The CoC provided after CHD treatment includes that the medical team maintains a good relationship with the patient during the patient’s hospitalization and provides the patient with individual education (diet, medications, activities, risk factors), cardiac rehabilitation exercises, and other related information [1,8,10]. CoC was applied to the cardiac rehabilitation of CHD patients, and the results showed that CoC can enhance patients’ prognosis and effectively improve the continuity of care information, management, and the relationship between the medical team and the patient [10].

The importance of CoC for chronic disease patients is emphasized in clinical care in Taiwan, and research has found that CoC can reduce the risk of patient readmission and improve their QoL [9]; related studies have also found that due to the high complexity of the disease of CHD patients, it is necessary to strengthen the CoC needs for patients from hospital to home as well as provide patient care-related information and appropriate nursing guidance during hospitalization in order to reduce the risk of sudden cardiac death [10]. The motivation of this study is for researchers to understand the CoC provided by the medical team to CHD patients during their hospitalization and whether CoC will affect their QoL. As previous research on CoC and the QoL of CHD patients is limited, this study adopted the continuity of care model, which intends to reduce the patient’s risk of disease and improve their QoL by understanding the relationship between CHD patients and the medical team during hospitalization, information transfer, and communication management with the medical team, and then, conduct follow-up tracking management [9]. Therefore, the purposes of this study are to (1) understand the sociodemographic characteristics, health status, continuity of care, and QoL of CHD patients; (2) explore the relationship between sociodemographic characteristics, health status, and continuity of care of CHD patients regarding QoL; and (3) explore the important predictive factors that affect the QoL for CHD patients.

## 2. Materials and Methods

### 2.1. Study Design and Participants

The Descriptive Correlational Design was adopted as the research design, and the data collection period was from 1 August 2019 to 16 July 2020. The collection site was located in the cardiac medical and surgical ward of a regional hospital in the central region of Taiwan, and convenience sampling was adopted. The ability to complete the structured self-filled questionnaires, or face-to-face interviews for CHD patients who were unable to read, and met the admission criteria, were adopted for the questionnaire survey. The researchers and a clinical nurse, who had received standardized training, conducted data collection on the days when the patients in stable condition were discharged. The inclusion criteria for this study were as follows: (1) patients with CHD diagnosed by a physician; (2) patients over 20 years old; (3) patients who can communicate in Mandarin or Taiwanese; (4) patients were willing to accept interviews, and can fill out the questionnaire by himself or herself or with the assistance of the researcher; (5) patients who agreed to participate in the research and signed the consent form. The exclusion criteria were those with vision or hearing impairment, dementia, or mental illness (such as depression, schizophrenia).

The number of samples in this study was estimated with G power 3.0.10 [11] statistical software, where the alpha value was 0.05, power was 0.8, and the effect size was set at 0.30 [8]. The calculated results estimated the minimum sample size of 108 patients. After the 20% possible loss rate (patients refusing to participate or withdrawing) was considered [12], the actual number of cases accepted in this study was 170. There were 7 invalid questionnaires, where questionnaires completed by patients themselves had too many missing values, and these were deleted. Finally, a total of 163 patients with coronary heart disease (CHD) were admitted for a participation rate of 95.88%.

### 2.2. Measurement

This study used a structured questionnaire for data collection. The research tools included 4 parts: sociodemographic characteristics of CHD patients, their health status, patient continuity of care scale, and quality of life scale.

#### 2.2.1. CHD Patients

Sociodemographic characteristics of CHD patients included age, gender, body mass index (BMI), marital status, living situation, religious belief, education level, employment status, economic status, discharge trends, etc.

#### 2.2.2. Health Status

This study was based on the number of diseases, time of illness, smoking, frequency of weekly exercises, treatment methods, whether they received Phase 1 cardiac rehabilitation, ADL, IADL, risk of disability, etc. ADL and IADL scales were used for the assessment of ADL functions. ADL is widely used at home and abroad, and the contents include the 10 items of eating, bathing, personal grooming and hygiene, dressing, urination control, stool control, movement when using the toilet, movement between chairs/beds, walking, and climbing stairs, with a score range of 0–100 points. To achieve good internal consistency, the Cronbach’s α should be 0.90 [13], and the ADL Cronbach’s α value of this study is 0.96. IADL is also widely adopted by relevant researchers around the world, and includes 8 types of abilities: going out, shopping on the street, washing clothes, cooking food, using the telephone, maintaining housework, taking medication, and handling finances, and each has a score ranging from 2 to 4 points. The lower the score, the worse the behavioral ability, and the total score is 24 points. A high score indicates the better independent ability of the patient. The literature review found that the retest reliability must reach 0.93 [14], and the IADL Cronbach’s α value of this study is 0.93. The risk of disability scale contains five domains: movement (5 items), nutrition (4 items), cognition (5 items), sociability (5 items), and depression (5 items), with a total of 24 items, which were scored by yes or no questions. In each domain, ≥1 point means a risk of disability, and the total score range is 0–24 points. The higher the score, the worse the risk of disability, as described in the domain [15]; the internal reliability the Cronbach’s alpha value of the scale should be 0.64 [9], and the Cronbach’s α value of the risk of disability scale in this study is 0.85.

#### 2.2.3. Patient Continuity of Care Scale

The original scale of the Patient Continuity of Care Questionnaire (PCCQ) was developed in 2008 [16] and later revised in 2017 [9] to meet the continuity of care scale in Chinese, as used in Taiwan. There are a total of 12 items, including 2 subscales: relationships with providers in hospital (5 items), and information transfer to patients (7 items). Each item is scored with a Likert 5-point scale, from 1 (Strongly disagree) to 5 (Strongly agree), and the total score range is 12–60 points. After the sum of the scores of each item was averaged, it became the score of each item. The PCCQ scale has good reliability and validity, and it has been pointed out the scale has good simultaneous validity and internal consistency [9], and the PCCQ Cronbach’s α value in this study was 0.97.

#### 2.2.4. Quality of Life Scale

This study used the Taiwanese concise version of the World Health Organization Quality of Life QoL Questionnaire (WHOQOL-BREF Taiwan Version) [17] for “Measuring Quality of Life”, which contains four domains: physiological health (7 items), psychological health (6 items), social relationships (4 items), and environment (9 items), as well as two local questions, for a total of 28 questions to evaluate the overall QoL. A Likert 5-point scale is adopted for scoring; the average score of each domain is 1–5 points, the score range of each domain is 4–20 points, and the scores in the four domains are added to represent the overall QoL score. The total score range is 16–80 points [17]. This study used the questionnaire as a measurement tool to determine the QoL of CHD patients. The original scale’s Content validity index (CVI) value was 0.90, and the internal consistency Cronbach’s α value was 0.95 [17]; the scale [9] adopted in this study showed good reliability (Cronbach’s α value = 0.93), and the Cronbach’s α value of the QoL scale in this study is 0.90.

### 2.3. Data Collection

Before this research was carried out, it was reviewed and approved by the Institutional Review Board (IRB) of the regional teaching hospital in the central region of Taiwan (No.: KTGH 10814), and it complied with the 1975 ethical code of the “Declaration of Helsinki”. After the acceptance of the hospital was obtained to conduct this research, the hospital’s nursing supervisor introduced the CHD patients who met the inclusion criteria, and the research purposes were explained to the subjects, in order to obtain their consent before the research consent form was signed and data collection began. The self-reported questionnaire survey was used for data collection; if the patient had difficulty reading the questionnaire, the researcher read the questions one by one and filled in the patient’s answers. It took about 20 minutes to complete each questionnaire, and a total of 163 patients were included.

### 2.4. Ethical Considerations

This study was reviewed and approved by the Institutional Review Board of the teaching hospital in the central region of Taiwan. With the consent of the hospital, the researcher explained the purposes of the research to the research subjects, and they signed the consent form before the questionnaire survey. During the questionnaire filling process, patients could withdraw from participating in the research at any time, and their treatment rights would never be compromised. The researchers collected data with strict confidentiality and the data of the subjects was coded; thus, there was no issue of information leaks to ensure the privacy of the research subjects.

### 2.5. Statistical Analyses

After the data were encoded, the Chinese version of SPSS for Window 25.0 (IBM Corp., Armonk NY, USA) was used for statistical analysis of the number of times, percentage, average, standard deviation, independent sample *t*-testing, Pearson product difference correlation, one-way ANOVA, multiple stepwise linear regression analysis, etc. The substitution of the variable mean was adopted to address missing values. After the QoL regression model was selected, the Kolmogorov–Smirnov (KS test) test was used to verify whether the residuals of the regression model conformed to normal distribution. For all statistical analyses, a *p*-value of <0.05 indicated that the estimate of the variable had statistical significance.

## 3. Results

There were 170 questionnaires sent out in this study, and a total of 163 questionnaires were returned for a recovery rate of 95.88%. The research results are explained as follows:

### 3.1. Overview of CHD Sociodemographic Characteristics, Health Status, Continuity of Care, and Quality of Life

The age distribution was between 20 and 97 years old, with an average age of 69.69 ± 13.62 years; 88 (54%) of the participants were women. The average age of male and female participants was 73.03 ± 12.41 and 66.85 ± 14.03, respectively. In terms of health status, the majority of the subjects coexisted with less than three diseases, with an average of 3.5 ± 1.37 diseases, and most patients were diagnosed with high blood pressure (73, 44.8%), diabetes (50, 30.7%), or angina pectoris (48, 29.4%) as the main diseases; the majority of patients had a disease history of 6 years or more (50.3%); most of the subjects (95, 58%) had the treatment method of heart catheterization; the ADL average score was 84.88 points ± 25.62 (moderately dependent), and their daily activity functions were better in eating, stool control, urination control, and putting on and taking off clothes; the average IADL score was 18.62 points ± 7.59) (total score 24 points), and better functions included using the phone, taking medications, and handling financial affairs (Table 1).

The mean of the QoL of CHD patients in this study was 56.56/80 ± 11.74, meaning a moderate QoL. The QoL in the social relationship domain was better, with an average of 15.02 ± 3.13, while the physiological health domain had the lowest score of 13.13 ± 2.80. The average QoL score of female participants was 58.31 ± 11.89, while that of male participants was 55.59 ± 12.87. Among the four domains of QoL, the three items with the lowest scores (the average score ranging from 1 to 5 points) were often having negative feelings (average 2.86 points), feeling that physical pain will hinder the need to do things (average 2.87 points), and consciously enjoying life (average 3.01 points); in terms of the risk of disability for CHD patients, it can be divided into five domains: movement, nutrition, cognition, sociability, and depression. The highest risk of disability was depression, with the average of 2.31 points ± 2.09; the average PCCQ score was 51.80/60 ± 6.04 (score index 86.33 points), the average score of the relationship with the medical team during hospitalization was 21.61 ± 2.63 (score index 86.44 points), and the average score of information transfer was 30.18 ± 3.71 (score index 86.23 points) (Table 2). Among the two domains of CoC, the three items with the lowest score (the average score ranging from 1 to 5 points) were receiving clear information that the disease may progress in the future (average 4.27 points), being told of disease-related dietary instructions (average 4.28), and feeling satisfied with the information provided by the medical team currently taking care of me (average 4.29). From the above results, it is known that the relationship and the information transfer between discharged CHD patients and the medical team must be strengthened in order that patients can receive adequate CoC.

### 3.2. Correlation between CHD Sociodemographic Characteristics, Health Status, Continuity of Care, and Quality of Life

The results of the sociodemographic characteristics show that the older the age, the better the QoL in the psychological domain (*r* = 0.175, *p* < 0.05), social relationships domain (*r* = 0.262, *p* < 0.01), and environmental domain (*r* = 0.231, *p* < 0.01); no significant difference was reached between gender and the four domains in QoL (*p* > 0.05); in terms of living situation, there was a significant difference between the living situation and the QoL in the four domains. The QoL of “living alone” was significantly better than that of “non-solitary people”; the QoL for the main source of income for patients and the environmental domain (*F* = 3.125, *p* < 0.05) both had a significant difference. Patients whose source of income was “children/spouse/siblings/parents” had better QoL than “pension/government grants” (Table 3).

In terms of health status, the higher the ADL and IADL scores of patients, the better their QoL in the physiological, psychological, social relationships, and environmental domains; in terms of the risk of disability, the risk of disability was significantly negatively correlated with the overall QoL, the CHD patients suffered from the risk of disability in movement, nutrition, social interaction, and depression; the fewer the risks of disability items, the better the QoL. Patient continuity of care and QoL were also positively correlated; when the patient’s relationship with the medical team during the hospitalization period and the CoC regarding information transfer were better, the overall QoL was higher (Table 3).

### 3.3. Important Predictors of CHD Quality of Life

Multiple stepwise linear regression analysis was used to determine the main factors that affected the QoL of CHD inpatients, and the factors that were statistically significantly related to the QoL, according to the results, were put into the regression model. Before stepwise analysis, the variables of the level of education, main source of income, etc. were converted into dummy variables, and the Kolmogorov–Smirnov test (*KS* = 0.92, *p* > 0.05) of the CHD QoL regression model was tested as a normal distribution; then, collinearity testing between the respective variables was carried out to determine the independence of the variables. The tolerance check results between the variables were all greater than 0.10, and the variance inflation factor (VIF) check results were all less than 10, which shows that there was no collinearity problem between the independent variables.

Analysis of the results of multiple stepwise linear regression shows that age, living situation, main source of income, IADL, depression in the domain of risk of disability, and information transfer in the domain of patient continuity of care were the best predictors of QoL. Among them, IADL had the greatest explanatory power, which can explain 26.1% of the variance, followed by age (18.1%), living situation (7%), information transfer (4.8%), main source of income from children/spouse/siblings/parents (1.8%), and depression in the domain of risk of disability (0.9%). The above variables can effectively explain 58.7% of the total variation of the overall QoL of CHD inpatients (F = 58.355, *p* < 0.001). The results show that the control of the social demographic characteristics, health status, risk of disability, and CoC of CHD inpatients had significant differences in their QoL (refer to Table 4).

## 4. Discussion

### 4.1. Current Quality of Life of CHD Patients and Status of Continuity of Care

This study found that the overall average QoL score of CHD patients was 56.56/80 (total score 100 points, score index 70.70 points), which was lower than the 81 points of CHD patients in ref [1] and higher than the 57.44 points of CHD patients in ref [7]. The reasons for the difference in the QoL may be due to different races, as the self-evaluation of the QoL of Caucasians is better than that of black or other races [1]. Gender difference may affect the QoL among CHD patients [18]. Compared with male CHD patients, elderly women have higher risks of overall cardiovascular diseases [18,19]. Smoking habits, diabetes, triglyceride, high-density lipoprotein (HDL), cholesterol levels, menopause, decreased estrogen, and psychosocial stressors (anxiety, depression, marital stress) all have great impacts on women’s cardiovascular system [19,20]. However, our research results did not find any significant difference between gender and QoL, which is consistent with the findings of Barham, Ibraheem, and Sa’ed [7]. The possible reason is that when women suffer from CHD, atypical symptoms of nausea, loss of appetite, and pain in jaw or neck would often occur; thus, female CHD patients should attach more importance to cardiovascular treatment and health care in order to maintain good QoL [21]. The QoL may also be affected by the severities of CHD, such as painful symptoms and less physical activity [22]. From the perspective of QoL, the average score in the physiological domain was the lowest (13.13 points), followed by the psychological domain with 13.72 points. According to the analysis results, CHD patients often have serious illnesses that lead to restrictions on physical health and functions, and they always feel fatigue, pain, or difficulty in sleeping [1,22], which restricts their independence in daily life functions (with difficulty in bathing, dressing, eating, walking, using the toilet, etc.). Thus, the QoL in this physiology domain is poor [6]. When CHD patients cannot relieve the physical symptoms and pain caused by chronic diseases on their own, it can easily cause feelings of powerlessness and negative psychological effects [23].

The results of this study show that the average scores of CoC for CHD patients were 4.27–4.36, while the average scores for CoC of chronic disease patients were 3.54–4.72, as also found in a previous survey [9]. According to analysis of the CoC of the two studies, the relationship between the patient and the medical team during hospitalization and the transmission of information were related to the patient’s QoL. When the patient perceived that the medical team understood his/her expectations and beliefs, felt confident that the current medical team would continue to take care of him/her after discharge from the hospital, and received information support, they had a better QoL. It is important for heart disease patients to receive related information on disease progression, disease symptoms, and post-discharge treatment [24]. Therefore, in order to clinically relieve the symptoms of CHD patients, in addition to improving the function and prognosis of CHD patients, it is necessary to strengthen the CoC by the medical team regarding the transmission of information to the patients so that the patients can know their own diagnosis, prescription drugs, and disease-related supportive information, such as diet instructions [25].

The CoC of CHD patients after discharge is a top priority, especially as the current health-care system is becoming more and more complex and fragmented [25]. Clinically, CoC relies on early screening by first-line care personnel when patients are admitted to the hospital, their creation of a complete physical and mental assessment, and referring to a case manager for discharge preparation services in a timely manner, in order for CHD patients to have good CoC [24]. Providing discharge preparation services during the patient’s hospitalization can improve communication and coordination between the patients, their family members, and inter-professional teams to facilitate patients receiving information on medication, rehabilitation, proper diet, physical activity, psychology, and society after they are discharged from the hospital. It can also enable patients to receive good care, resources, and services from community care workers after they return home from the hospital [25].

### 4.2. Factors Affecting Quality of Life of CHD Patients

Regarding the demographic characteristics of older CHD patients, this study found that CHD patients living alone and CHD patients whose main source of income were from children/spouse/siblings/parents were predictors of QoL. The older the age, the better the evaluation of their own psychological, social relationships, and environmental QoL, which can explain 18.1% of the variance. While this result is the same as some research results [17], it is inconsistent with previous literature [7]. The lower QoL of younger patients with CHD may be due to younger patients lack of information related to the disease, as provided by the medical professional team, coupled with the need to face specific problems, such as work, childbirth, social isolation, finances, etc.; thus, younger patients with CHD are prone to psychological phenomena, such as depression, anxiety, and stress, which reduces their QoL [22]. Older patients may be more able to accept the disease and follow the instructions of the medical team to receive treatment due to the longer duration of the disease; therefore, such patients have a better evaluation of their QoL [26]. This study also found that the QoL of patients living alone was better, and this result is consistent with previous literature [27]; the possible reason is that people who live alone receive more support from their interactions with siblings, friends, and neighbors, while those divorced or widowed and living alone have a better chance of getting satisfaction from their adult child or grandchild, which prevents the patients from feeling neglected. Related research also shows that if a patient living alone after being discharged from the hospital has a medical team to provide individualized community services at home, such as housework support (food preparation), home environment maintenance and service, sports support, connection support (hygiene products), emergency services, and other supportive service, it will allow patients to experience a better QoL [28].

With the control of other factors, CHD patients with the main source of income from children/spouses/brothers/sisters/parents had better evaluations of their QoL than those from pension/government grants, which can predict the QoL of CHD patients and explain 7% of the variance. The results of this study are consistent with previous studies [29]. When CHD patients are financially supported by adult children, this may bring them a sense of security, which has a positive impact on their QoL [30].

Another finding of this study is that the patients who can go home, rather than being transferred to an acute or chronic ward after being discharged from the hospital, had better evaluation of their QoL; related research results show that most patients are eager to go home to recuperate, and when patients in hospital receive (enough) information about the disease and disease progress, medication, rehabilitation, and psychosocial aspects, it will help them to obtain better QoL [31]. Therefore, in order to improve the QoL of patients, the medical professional team needs to emphasize the evaluation, planning, information, and education of patients before discharge, strengthen their abilities in self-management, and ensure that patients and main caregivers understand the importance of home environment preparation to improve their QoL [1,31].

The related variables of health status help to examine the QoL of CHD patients. The results of this study show that ADL, IADL, and Risk of disability (Movement, Nutrition, Sociability, Depression) are related to the QoL. Under the circumstances of controlling other variables, the research results show that the better the ADL and IADL of the patients, the better their evaluation of their QoL, and this result is consistent with previous literature [5]; the possible cause is the various symptoms caused by the disease (such as dyspnea, fatigue, pain), disability, etc., will cause the patient’s ADL and IADL to be restricted (that is, bathing, dressing, using the toilet, moving, going out, shopping, and cooking food), their life independence to be impaired, their social participation ability to be reduced, and their dependence on care to be increased, which affects the patient’s QoL [5,6]. Regression analysis found that IADL and Risk of disability (Depression) were the main predictors of QoL, which can increase the variance explanation of QoL by 26.1% and 0.9%, respectively. IADL is the most predictive factor affecting the QoL of patients with CHD. Therefore, in addition to maintaining ADL in patients with CHD, the promotion of IADL should not be ignored [6]. Related studies found that IADL dysfunction in CHD patients may directly affect the interactions between the patient and the neighborhood, reduce social activities, make the patient feel lonely, and significantly increase the severity of symptoms, thereby reducing the QoL of the patient [6]. Therefore, in order to improve the IADL of patients, it is very important to plan patient health care and activity promotion. The medical professional team can also advocate government policies to encourage patients to participate in long-term care 2.0 for delayed disability activities, including muscle strengthening exercises, dietary nutrition, life function reconstruction training, social participation, etc., to improve the QoL of patients [32]. On the other hand, the results show that a significant negative correlation exists between the risk of disability (movement, nutrition, sociability, and depression) and QoL and among the dimensions of risk of disability, where depression has the highest score for risk of disability; thus, depression in the domain of risk of disability is a predictor for the QoL of patients. The QoL of CHD patients was affected by depression [23]; the impact of disability risk will cause patients to face obstacles in health and rehabilitation services. Depression has also been related to the mortality of CHD patients, which increased the risk of CHD by 1.64 times, thereby reducing the QoL [33]. Therefore, medical professional teams should provide CHD patients with better disease management during the patient’s hospitalization; they should prevent hypofunction, increase patients’ acceptance, and encourage their confidence in dealing with their disease, as this can delay depression and the deterioration of QoL [27].

Another feature of this study is the inclusion of variables related to the CoC of patients, as such variables help to examine the impact of CoC on the QoL of CHD patients. According to regression analysis, information transmission by the medical team to patients regarding the CoC can predict the QoL of CHD patients and explain 4.8% of the variance. The results show that the better the quality of information that patients receive during hospitalization (clear diagnosis, receiving clear information about the progress of the disease, emergency contact information, medication, diet, etc.), the better their QoL. The results of this study will allow medical teams to understand that CHD patients’ receiving information during their hospitalization directly affects their QoL [1,24]. Therefore, in order to improve the QoL of CHD patients, it is necessary for their medical team to strengthen the CoC for patients during hospitalization, especially in terms of information transfer, and to provide patients with sufficient information that affects their personal health or follow-up care to improve their QoL [24,26].

## 5. Conclusions

Overall, this study found that the QoL of CHD patients was a moderate QoL. Age, living situation, main source of finance, IADL, risk of disability, and CoC were predictors that affect the QoL of CHD patients, which can explain 58.7% of the total variance. In order to improve the QoL of CHD patients, it is recommended that medical teams must strengthen the patient’s IADL functions; encourage patients to participate in community health promotion activities; include depression in the risk of disability as an indicator in the evaluation of CHD patients; and enable them to obtain the information required to deal with their disease, in order that patients can receive good CoC. The government should also strengthen the financial protection of patients and create a forward-looking and comprehensive social welfare insurance system plan to promote their physical and psychological health and improve their QoL.

Limitations and Suggestions: This study was limited to a certain regional hospital in central Taiwan; thus, it is difficult to make comprehensive inferences. It is recommended that a more rigorous and effective evaluation system can be created with an experimental design that focuses on CoC intervention to effectively strengthen the cooperation of professional medical teams. In-depth discussions of the CoC and QoL of CHD patients can be used as a reference for long-term care intervention research. The research results can also be applied to clinical practice for medical professional teams to understand the factors that affect the QoL of CHD patients, which is helpful for improving the QoL of CHD patients.

## Figures and Tables

**Table 1:** Social characteristics and health status of subjects (*N* = 163).

Variables		*N*	%
Sociodemographic Characteristics			
Age	<65 >65	52 111	31.9 68.1
Mean (SD)	69.69 (13.62)		
Gender	Male	88	54
	Female	75	46
BMI (kg/m^2^)	Mean (SD) 25.60 (4.06)		
	<18.5	1	0.6
	18.5 ≤ BMI < 24	56	34.4
	24 ≤ BMI < 27	60	36.8
	27 ≤ BMI < 30	33	20.2
	30 ≤ BMI < 35	11	6.7
	≥35	2	1.2
Marital status	Single (unmarried/divorced/widowed)	61	37.4
	Spouse (married/cohabiting, separated)	102	62.6
Living situation	Solitary	33	20.2
	Not alone	130	79.8
Number of people living in household	0	33	20.2
	<3	76	46.7
	>3	54	33.1
Mean (SD)	2.61 (1.99)		
Persons living with	Spouse	85	52.1
	Children	90	55.2
	Grandchildren/parents/brothers and sisters/friends/foreign domestic helpers	60	36.8
Religion	No	35	21.5
	Yes	128	78.5
Level of education	Illiterate/literate (self-study)/primary	83	50.9
	Junior high school/junior/high school (vocational)	53	32.5
	Junior college and above	27	16.6
Employment status	Unemployed	113	69.3
	Employed	50	30.7
Income	Sufficient/more than sufficient	27	16.6
	Roughly enough	98	60.1
	Slightly insufficient/inadequate	38	23.3
Main source of income	Children/spouse/brothers or sisters/parents	96	85.9
	Pension/ government grants	36	22.1
	Employment	31	19.0
Discharge trend	Go home	158	96
	Transfer to acute/chronic ward	5	3.1
Health status			
Number of diseases	<3	82	50.3
	3–5	68	41.7
	6 and above	13	8.0
Mean (SD)	3.5 (1.37)		
Time since diagnosis	<1 year	46	28.2
	1–5 years	53	32.5
	6 years and above	64	39.3
Smoking habit	Without	110	67.5
	With	53	32.5
Frequency of weekly exercise	Never	45	27.6
	<3 times a week	85	52.1
	>3 times a week	33	20.2
Treatment method	Cardiac catheterization	95	58.3
	Angioplasty and vascular stenting	63	38.7
	Coronary artery bypass graft	5	3.1
Phase−1 cardiac rehabilitation	Without	119	73
	With	44	27
ADL	Mean (SD) 84.88 (25.62)		
IADL	Mean (SD) 18.62 (7.59)		

BMI: body mass index, ADL: activities of daily living, IADL: Instrumental activities of daily living, SD: standard deviation.

**Table 2:** World Health Organization Quality of Life QoL Questionnaire (WHOQOL–BREF) Taiwan version, Patient Continuity of Care Questionnaire (PCCQ) scores and risk of disability in patients with coronary heart disease.

**Variables**	**Mean Score (SD)**	**Mean/Item** **(SD)**	**Score Indicator**
WHOQOL–BREF Taiwan Version Total QoL score (16–80)	56.56 (11.74)	3.54 (0.73)	70.70
Physiological health domain (4–20)	13.13 (2.80)	3.28 (0.70)	65.65
Psychological health domain (4–20)	13.72 (3.48)	3.43 (0.87)	68.60
Social relationships domain (4–20)	15.02 (3.13)	3.75 (0.78)	75.10
Environment domain (4–20)	14.70 (3.14)	3.67 (0.78)	73.50
PCCQ			
Total score of PCCQ (12–60)	51.80 (6.04)	4.32 (0.50)	86.33
Relationships with providers during hospitalization (5–25)	21.61 (2.63)	4.32 (0.53)	86.44
Information transfer to patients (7–35)	30.18 (3.71)	4.31 (0.53)	86.23
**Variables**	**Mean Score (SD)**	***n***	***%***
Coronary heart disease risk of disability (24)	9.36 (6.23)		
Movement (5)	1.98 (1.63)	122	74.8
Nutrition (4)	1.86 (1.52)	121	74.2
Cognition (5)	1.85 (1.45)	124	76.1
Sociability (5)	1.37 (1.86)	72	44.2
Depression (5)	2.31 (2.09)	112	68.7

The total score of each QoL domain ranges from 4 to 20 points, where a higher score indicates better QoL in that domain. WHOQOL: World Health Organization quality of life, SD: standard deviation. QoL: quality of life, PCCQ: Patient Continuity of Care Questionnaire. A score of 1 or above in each subscale shows the disability risk in that domain. Mean/item: the sum of scores of all items/total number of items (the average score of the items in QoL and PCCQ ranges from 1 to 5 points).

**Table 3:** Correlation between sociodemographic characteristics, health status, risk of disability, patient continuity of care, and QoL among patients with coronary heart disease (*N* = 163).

Variables	Physiological Health	Psychological	Social	Environment
Gender ^a^	*t* = 0.539	*t* = 1.871	*t* = 1.799	*t* = 0.862
Marital status ^a^	*t* = 1.470	*t* = 1.776	*t* = 0.923	*t* = 2.754 **
① Single(unmarried/divorced/widowed)				
② Spouse (married/cohabiting, separated)				
Living situation ^a^	*t* = 6.442 ***	*t* = 4.682 ***	*t* = 4.568 ***	*t* = 3.504 ***
① Solitary				
② Not alone				
Religion ^a^	*t* = 2.943 **	*t* = 2.596 *	*t* = 1.987 *	*t* = 1.452
① Without				
② With				
Employment status ^a^	*t* = −2.219 *	*t* = −1.374	*t* = −2.960 **	*t* = −0.481
① Unemployed				
② Employed				
Discharge trend ^a^	*t* = 1.383 **	*t* = 2.924 **	*t* = 2.518 *	*t* = 2.190 *
① Go home ② Transfer to acute/chronic ward				
Smoking habit ^a^	*t* = −3.354 **	*t* = −3.066 **	*t* = −2.608 **	*t* = −1.178
① Without ② With				
Level of education ^b^	*F* = 7.553 ***	*F* = 6.676 **	*F* = 4.862 **	*F* = 2.950
① Illiterate/literate (self- study)/Primary				
② Junior high school/junior/high school (vocational)				
③ Junior college and above	③ > ①	③ > ①		
Scheffe post-comparison	③ > ②	③ > ②	③ > ②	
Main source of income ^b^	*F* = 1.329	*F* = 2.055	*F* = 3.125*	*F* = 2.236
① Children/spouse/brothers or sisters/parents				
② Pension/government grants				
③ Employment				
Scheffe post-comparison			① > ②	
Age	*r* = 0.137	*r* = 0.175 *	*r* = 0.262 **	*r* = 0.231 **
Number of people living in household	*r* = −0.249 ***	*r* = −0.246 **	*r* = −0.212 **	*r* = −0.179 *
ADL	*r* = 0.381 ***	*r* = 0.355 ***	*r* =0.298 ***	*r* =0.240 **
IADL	*r* = 0.521 ***	*r* = 0.522 ***	*r* =0.451 ***	*r* =0.516 ***
Risk of disability				
Overall	*r* = −0.081	*r* = −0.049	*r* = −0.040	*r* = −0.067
Movement	*r* = −0.263 ***	*r* = −0.248 ***	*r* = −0.224 **	*r* = −0.156 *
Nutrition	*r* = −0.204 **	*r* = −0.233 **	*r* = −0.211 **	*r* = −0.185 *
Cognition	*r* = −0.028	*r* = −0.035	*r* = −0.012	*r* = −0.009
Sociability	*r* = −0.448 ***	*r* = −0.467 ***	*r* = −0.358 ***	*r* = −0.341 ***
Depression	*r* = −0.218 ***	*r* = −0.221 **	*r* = −0.183 *	*r* = −0.175 *
PCCQ				
Overall	*r* = 0.329 ***	r = 0.421 ***	*r* = 0.421 ***	*r* = 0.359 ***
Relationships with providers during hospitalization	*r* = 0.321 ***	*r* = 0.399 ***	*r* = 0.417 ***	*r* = 0.346 ***
Information transfer to Patients	*r* = 0.308 ***	*r* = 0.402 ***	*r* = 0.390 ***	*r* = 0.399 ***

* *p* < 0.05, ** *p* < 0.01, *** *p* < 0.001. ^a^: *t*-test, ^b^: *F*-test, ADL: activities of daily living, IADL: instrumental activities of daily living, PCCQ: Patient continuity of care questionnaire.

**Table 4:** Correlations between sociodemographic characteristics, health status, risk of disability, continuity of care, and quality of life among patients with coronary heart disease (N = 163).

Variables	QoL						
	B	SE	Beta	Adjust R^2^	*t*	95%CI	*p*
Age	0.281	0.054	0.307	0.181	5.247	(0.175, 0.387)	0.001 ***
Living situation	−7.086	1.877	−0.299	0.07	−3.775	(−3.378, −1.794)	0.001 ***
Main source of income							
Pension/government grants	−4.210	1.526	−0.140	0.018	−2.758	(−7.224, −1.195)	0.007 **
Children/spouse/brothers or sisters/parents (reference group)							
Health status							
IADL	0.923	0.098	0.562	0.261	9.378	(0.729, 1.117)	0.001 ***
Risk of disability							
Depression	−0.725	0.354	−0.122	0.009	−2.048	(−1.424, −0.026)	0.042 *
PCCQ							
Information transfer to patients	0.752	0.179	0.244	0.048	4.212	(0.399, 1.105)	0.001 ***

Linear regression was used for data analysis. B: unstandardized regression coefficient, IADL: instrumental activities of daily living, PCCQ: patient continuity of care questionnaire, t: if the p-value obtained from the regression coefficient is <0.05, the dependent variable can be effectively predicted using independent variables, adjust R^2^: 0.587, *F*: 58.355, * *p* < 0.05, ** *p* < 0.01, *** *p* < 0.001.
